# Sulfur-Mediated-Alleviation of Aluminum-Toxicity in *Citrus grandis* Seedlings

**DOI:** 10.3390/ijms18122570

**Published:** 2017-12-03

**Authors:** Peng Guo, Qiang Li, Yi-Ping Qi, Lin-Tong Yang, Xin Ye, Huan-Huan Chen, Li-Song Chen

**Affiliations:** 1 Institute of Plant Nutritional Physiology and Molecular Biology, College of Resources and Environment, Fujian Agriculture and Forestry University (FAFU), Fuzhou 350002, China; 2140807001@fafu.edu.cn (P.G.); 1160807008@fafu.edu.cn (Q.L.); talstoy@fafu.edu.cn (L.-T.Y.); yexin1000@fafu.edu.cn (X.Y.); 1170807011@fafu.edu.cn (H.-H.C.); 2 Institute of Materia Medica, Fujian Academy of Medical Sciences, Fuzhou 350002, China; qiyiping2008@hotmail.com; 3 Fujian Provincial Key Laboratory of Soil Environmental Health and Regulation, College of Resources and Environment, FAFU, Fuzhou 350002, China; 4 The Higher Education Key Laboratory of Fujian Province for Soil Ecosystem Health and Regulation, College of Resources and Environment, FAFU, Fuzhou 350002, China

**Keywords:** aluminum-toxicity, antioxidant enzymes, *Citrus grandis*, photosynthesis, sulfur metabolism

## Abstract

Limited data are available on the sulfur (S)-mediated-alleviation of aluminum (Al)-toxicity in higher plants. *Citrus grandis* seedlings were irrigated for 18 weeks with 0.5 mM MgSO_4_ or 0.5 mM MgSO_4_ + 0.5 mM Na_2_SO_4_, and 0 (−Al) or 1 mM AlCl_3_·6H_2_O (+Al, Al-toxicity). Under Al-toxicity, S decreased the level of Al in leaves; increased the relative water content (RWC) of roots and leaves, the contents of phosphorus (P), calcium (Ca) and magnesium (Mg) per plant, the dry weights (DW) of roots and shoots, the ratios of root DW/shoot DW, and the Al-induced secretion of citrate from root; and alleviated the Al-induced inhibition of photosynthesis via mitigating the Al-induced decrease of electron transport capacity resulting from the impaired photosynthetic electron transport chain. In addition to decreasing the Al-stimulated H_2_O_2_ production, the S-induced upregulation of both S metabolism-related enzymes and antioxidant enzymes also contributed to the S-mediated-alleviation of oxidative damage in Al-treated roots and leaves. Decreased transport of Al from roots to shoots and relatively little accumulation of Al in leaves, and increased leaf and root RWC and P, Ca, and Mg contents per plant might also play a role in the S-mediated-alleviation of Al-toxicity.

## 1. Introduction

Aluminum (Al) exists mainly as the forms of insoluble deposits when soil is neutral or mildly acidic, but is released from these deposits into soil solution when soil has a pH < 5.0. The micromolar concentration of Al^3+^ can inhibit root growth, thus reducing water and nutrient uptake and crop yield. Therefore, Al-toxicity is the major factor limiting plant growth and production in many acidic soils that account for over 50% of the world’s potentially cultivated lands. Furthermore, soil pH is rapidly declining [1,2,3,4].

Plants survive in acidic soils with high levels of active Al via two main mechanisms of Al detoxifications: (a) external detoxification, including the Al-induced release of organic acid (OA) anions from roots; and (b) internal detoxification [5,6,7,8]. Al-toxicity can cause the accumulation of reactive oxygen species (ROS), thus leading to peroxidation of proteins and membrane lipids in plant cells. Lipid peroxidation may cause loss of plasma membrane integrity and electrolyte leakage [9,10,11,12,13]. Increasing evidence shows that antioxidant enzyme activities play a key role in plant Al-tolerance [14,15,16]. The Al-sensitive sorghum line displayed higher ROS levels in the root tips than the Al-tolerant line did [17]. Also, more antioxidant enzymes were induced to higher levels in the Al-tolerant sorghum line than those in the Al-sensitive line root tips [15]. The abundances of proteins related to ROS scavenging were increased by Al in Al-tolerant *Citrus sinensis* and Al-intolerant *Citrus grandis* roots and leaves, with a greater increase in *C. sinensis* roots and leaves [18,19]. Genes involved in ROS scavenging were upregulated by Al-toxicity in *C. sinensis* and *C. grandis* roots, especially in *C. sinensis* roots [20].

Sulfur (S)-containing compounds synthesized during S metabolism, including reduced glutathione (GSH), H_2_S, and cysteine (Cys), play crucial roles in the detoxification of Al and heavy metals and the mitigation of oxidative stress [21,22]. Limited studies showed that S could alleviate Al-toxicity of barley [9,23], wheat [24], and oilseed rape [25] via increasing antioxidant capability and decreasing ROS and malondialdehyde (MDA) levels. The decreased uptake of Al, increased uptake of phosphorus (P), calcium (Ca), and magnesium (Mg), and enhanced Al-induced secretion of citrate from roots might also contribute to the S-mediated alleviation of barley Al-toxicity [9,23]. All these studies, however, have focused on herbaceous plants. *Citrus* belong to woody fruit trees that display poor growth and a shortened lifespan when grown in acidic soils with high active Al [26]. In China, *Citrus* are planted mainly in acidic and strong acidic soils. In 2011, we investigated the pH of 319 soil samples from *C. grandis* orchards, situated in Pinghe, Zhangzhou, Fujian, China. Over 90% of soils had a pH < 5.0 [3]. Jiang et al. isolated more S metabolism-related proteins increased in abundance from Al-treated *C. sinensis* roots than those from Al-treated *C. grandis* roots [18]. Li et al. obtained similar results from Al-treated *C. sinensis* and *C. grandis* leaves [19]. Recently, Guo et al. found that Al-toxicity increased S levels in *C. sinensis* roots and leaves and *C. grandis* roots, but not in *C. grandis* leaves, and S levels were higher in *C. sinensis* roots and leaves than those in *C. grandis* roots and leaves with or without Al-stress, with the exception that the root S level was similar between the two *Citrus* species without Al-stress. Genes involved in S uptake and export were upregulated and downregulated by Al in *C. sinensis* roots, respectively [20]. Thus, *Citrus* Al-toxicity should be alleviated by S.

Here, we first investigated the effects of Al and S interactions on growth and Al-induced secretion of OA anions from roots; elements, relative water content (RWC), H_2_O_2_ production, electrolyte leakage, total soluble proteins, MDA, antioxidants, and antioxidant enzymes, and S metabolism-related enzymes in roots and leaves; and gas exchange, pigments, ribulose-1,5-bisphosphate carboxylase/oxygenase (Rubisco), and photosynthetic electron transport in leaves of Al-intolerant *C. grandis* seedlings. The objective was to establish the role of S in alleviating *Citrus* Al-toxicity at the physiological level.

## 2. Results

### 2.1. Seedling Growth, Root, and Leaf Elements

**Figure 1:**
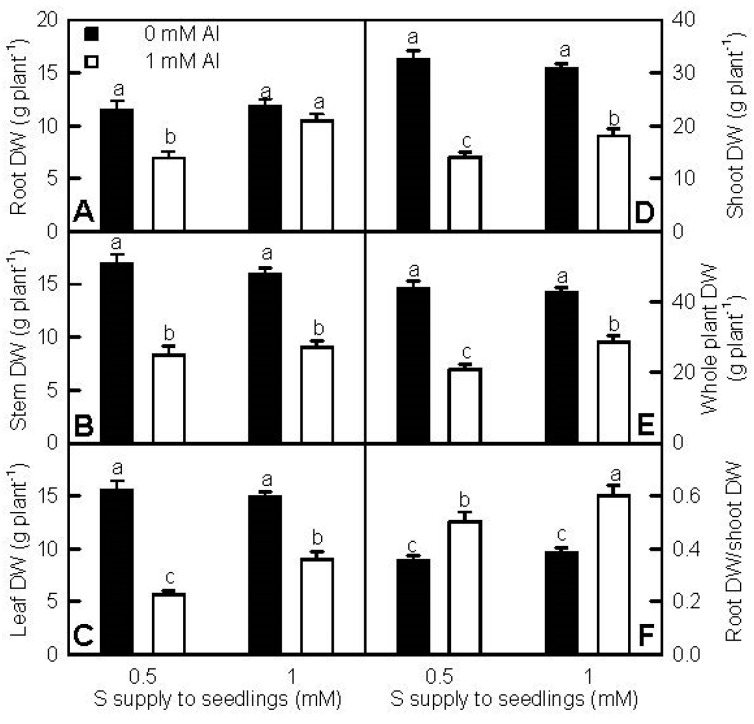
Effects of sulfur(S) and aluminum(Al) interactions on root (**A**), stem (**B**), leaf (**C**), shoot (**D**), and whole plant (**E**) dry weight(DW), and root DW/shoot DW ratio (**F**) in *C. grandis* seedlings. Bars represent means ± SE (*n* = 15). Differences among the four treatments were analyzed by two (Al levels) × two (S levels) ANOVA. Different letters indicate a significant difference at *p* < 0.05.

To examine the alleviation of S on Al-toxicity in *C. grandis* seedlings, the effects of S and Al interactions on seedling growth were investigated (Figure 1 and Figure S1). Al-toxicity decreased plant height, root, stem, leaf, shoot, and whole plant dry weights (DW), and increased the root DW/shoot DW ratio, with the exception that root DW was similar between the two Al treatments at 1 mM S. All these parameters did not significantly differ between the two S treatments at the absence of Al, but were higher at 1 mM S than those at 0.5 mM S under Al-toxicity. The only exception was that stem DW was similar between the two S treatments under Al-toxicity. These results indicated that S alleviated the Al-induced inhibition of growth.

**Figure 2:**
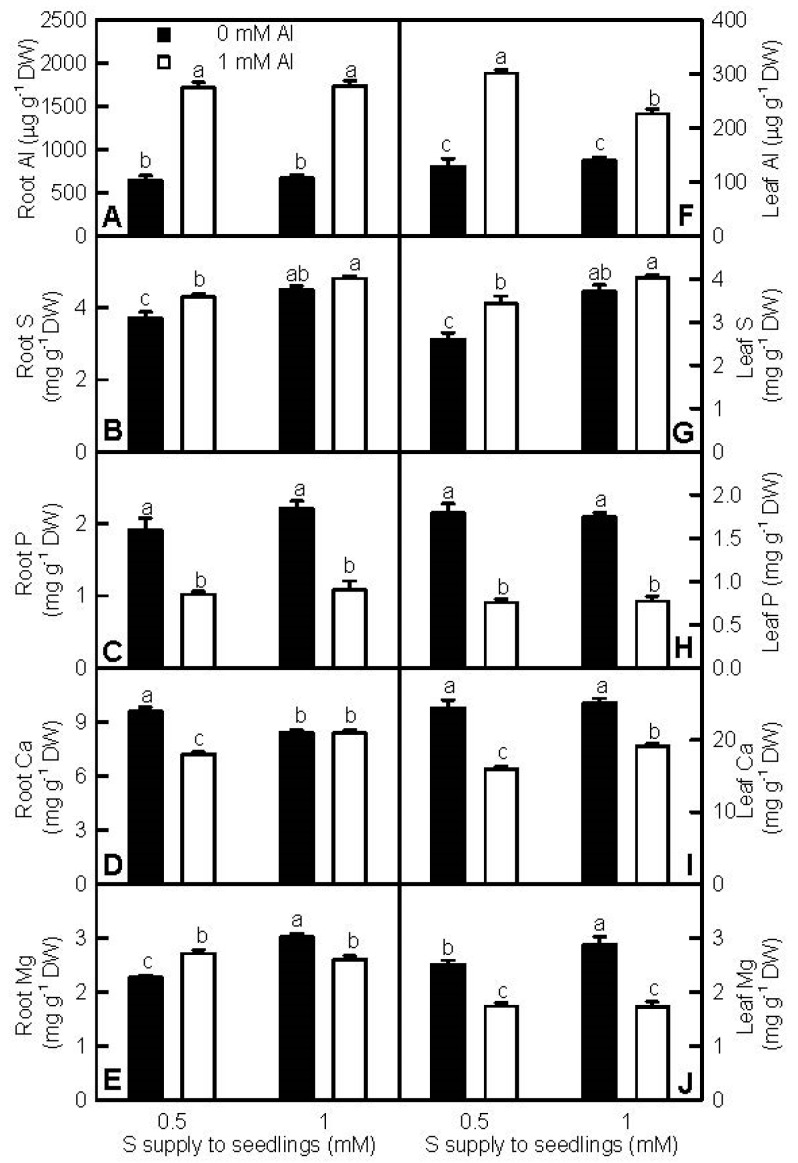
Effects of S and Al interactions on Al (**A**,**F**), S (**B**,**G**), P (**C**,**H**), Ca (**D**,**I**), and Mg (**E**,**J**) concentrations in roots (**A**–**E**) and leaves (**F**–**J**). Bars represent means ± SE (*n* = 4–8). Differences among the four treatments were analyzed by two (Al levels) × two (S levels) ANOVA. Different letters indicate a significant difference at *p* < 0.05.

To explore the effects of S and Al interactions on Al accumulation in roots and leaves, we measured Al, S, P, Ca, and Mg levels in roots and leaves (Figure 2). Al-toxicity increased or did not affect root and leaf Al and S levels, but decreased or did not affect root and leaf P, Ca, and Mg levels, with the exception that Mg level in 0.5 mM S-treated roots was increased by Al-toxicity. S increased or did not alter Al, S, P, Ca, and Mg levels in roots and leaves, with the exceptions that Al level in +Al leaves was lower at 1 mM S than that at 0.5 mM S, and that Ca level in −Al roots was lower at 1 mM S than that at 0.5 mM S.

### 2.2. Leaf Gas Exchange, Rubisco, and Pigments

To verify the role of S in alleviating the Al-induced inhibition of photosynthesis, we tested the effects of Al and S interactions on leaf exchange and the activity of Rubisco, a key enzyme in photosynthesis (Figure S2). Leaf CO_2_ assimilation, stomatal conductance (Gs), transpiration rate, and Rubisco activity were similar between the two S treatments at the absence of Al, but lower at 0.5 mM S than those at 1 mM S under Al-toxicity. Intercellular CO_2_ concentration (Ci) did not differ among the four treatment combinations.

Next, we investigated the effects of S and Al interactions on photosynthetic pigments (Figure S3). Leaf concentrations of chlorophyll (Chl) a, Chl b, Chl (a+b), and carotenoids (Car) were similar between the two S treatments at the absence of Al, but lower at 0.5 mM S than those at 1 mM S under Al-toxicity.

### 2.3. Leaf Chl a Fluorescence

**Figure 3:**
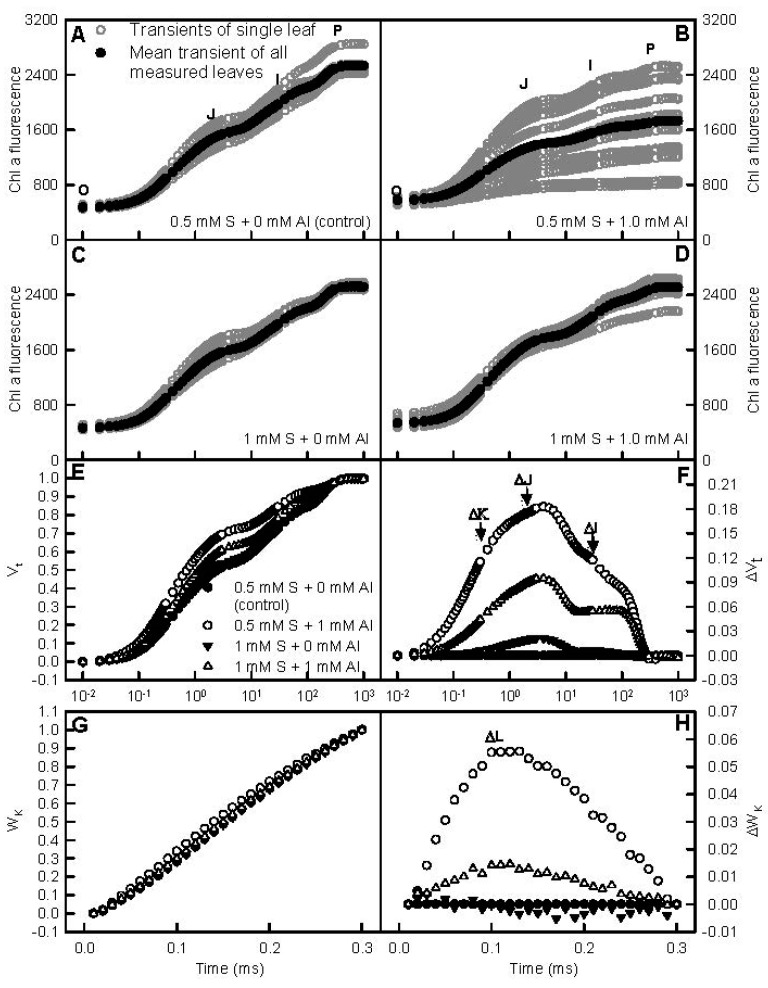
Effects of S and Al interactions on high irradiance actinic-light-induced OJIP transients of dark-adapted leaves (**A**–**D**) and the different expressions derived from the mean transients in dark-adapted leaves: (**E**) between F_o_ and F_m_: V_t_ = (F_t_ − F_o_)/(F_m_ − F_o_) and (**F**) the differences of the four samples to control; (**G**) between F_o_ and F_300μs_: W_K_ = (F_t_ − F_o_)/(F_300μs_ − F_o_) and (**H**) the differences of the four samples to control.

We examined the effects of S and Al interactions on leaf Chl a fluorescence in order to understand the mechanism by which S alleviated Al-induced inhibition of leaf CO_2_ assimilation (Figure 3 and Figure S4). S had little influence on Chl a fluorescence (OJIP) transients in −Al leaves. The heterogeneity of samples was increased by Al-toxicity at 0.5 mM S, but almost unaffected at 1 mM S. Compared with controls, Al-treated leaves displayed positive ΔL-, ΔK-, ΔJ-, and ΔI-steps, which were more pronounced at 0.5 mM S than those at 1 mM S (Figure 3).

The Al-induced alterations of all 20 fluorescence parameters were more pronouncedat 0.5 mM S than those at 1 mM S. At the absence of Al, all these parameters were similar between the two S treatments. Under Al-toxicity, minimum fluorescence (F_o_), approximated initial slope (in ms^−1^) of the fluorescence transient V = f(t) (M_o_), specific energy fluxes per reaction center (RC) for absorption (ABS/RC), specific energy fluxes per RC for energy dissipation (DI_o_/RC), quantum yield for energy dissipation (DI_o_/ABS), and non-photochemical deexcitation rate constant (K_N_) were higher at 0.5 mM S than those at 1 mM S, but the reverse was the case for the other 14 parameters (Figure S4).

### 2.4. Al-Induced Secretion of Malate and Citrate

The Al-induced secretion of root OA anions is the best recorded mechanism of Al-tolerance in higher plants [5,8]. As shown in Figure S5, the Al-induced secretion of citrate and malate from +Al excised roots was higher than that from −Al excised roots. Interestingly, the Al-induced secretion of citrate from 1 mM S + 1 mM Al-treated excised roots was higher than that from 0.5 mM S + 1 mM Al-treated excised roots. However, S did not affect the Al-induced secretion of malate and citrate from −Al excised roots and of malate from +Al excised roots.

### 2.5. RWC, H_2_O_2_ Production, MDA, Electrolyte Leakage, and Total Soluble Proteins in Roots and Leaves

**Figure 4:**
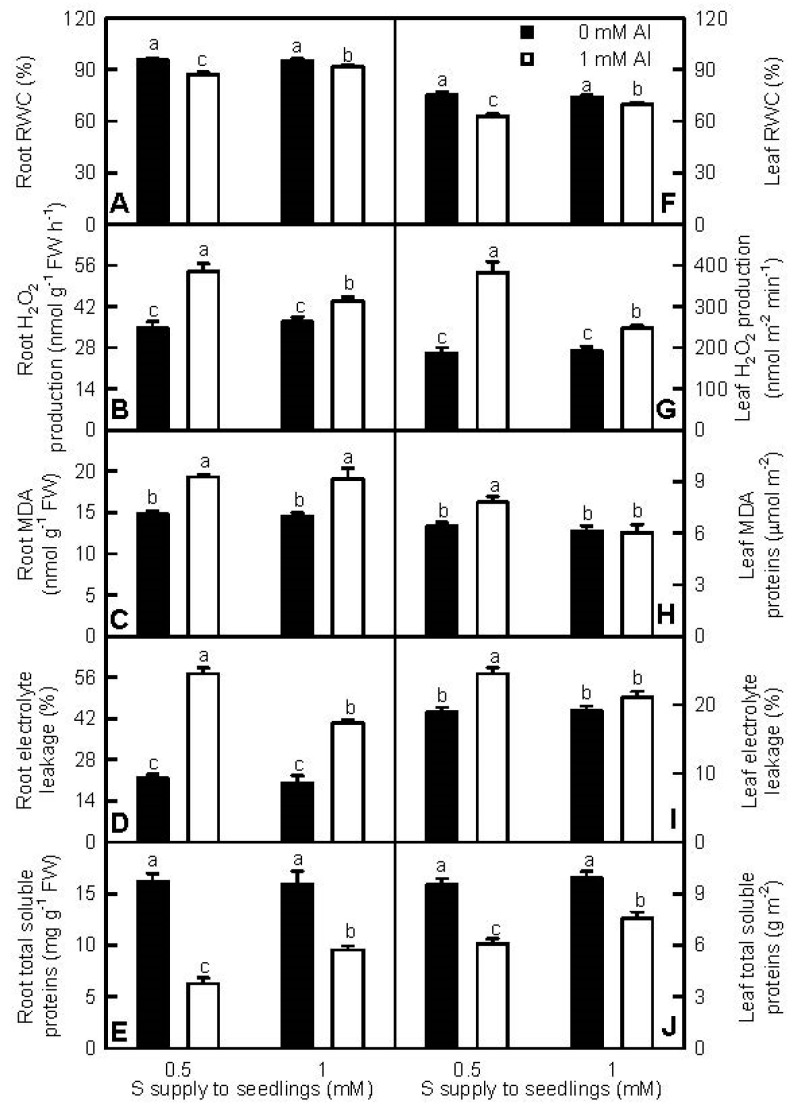
Effects of S and Al interactions on relative water content (RWC) (**A**,**F**), H_2_O_2_ production (**B**,**G**), malondialdehyde (MDA) concentrations (**C**,**H**), electrolyte leakage (**D**,**I**), and total soluble protein concentrations (**E**,**J**) in roots (**A**–**E**) and leaves (**F**–**J**). Bars represent means ± SE (*n* = 4). Differences among the four treatments were analyzed by two (Al levels) × two (S levels) ANOVA. Different letters indicate a significant difference at *p* < 0.05.

To investigate the role of S in alleviating the Al-induced inhibition of water uptake, we measured RWC in roots and leaves (Figure 4A,F). At 0 mM Al, root and leaf RWC were similar between the two S treatments. Under Al-toxicity, root and leaf RWC were higher at 1 mM S than those at 0.5 mM S.

Al-toxicity can stimulate ROS production, thus causing peroxidation of proteins and membrane lipids in plant cells [10,11,12,13]. Both MDA (an indicator for lipid peroxidation) and electrolyte leakage (an indicator for cell membrane integrity) are often used to estimate the ROS-mediated damage to cell membranes and to evaluate plant Al-tolerance [12]. To explore the role of S in mediating Al-induced oxidative stress, we measured H_2_O_2_ production, MDA, and total soluble protein levels and electrolyte leakage in roots and leaves (Figure 4B–E,G–J). Al-toxicity decreased total soluble protein levels and increased H_2_O_2_ production, MDA levels, and electrolyte leakage in roots and leaves, with the exceptions that leaf MDA level and electrolyte leakage were not altered by Al-toxicity at 1 mM S. At 0 mM Al, the four parameters were similar between 1 mM and 0.5 mM S-treated roots and leaves. Under Al-toxicity, total soluble protein levels in roots and leaves were higher at 1 mM S than those at 0.5 mM S, but the reverse was the case for H_2_O_2_ production, MDA levels, and electrolyte leakage in roots and leaves, with the exception that MDA level was similar between 1 mM and 0.5 mM S-treated roots. 

### 2.6. Antioxidant Enzymes, S Metabolism-Related Enzymes, and Antioxidants

**Figure 5:**
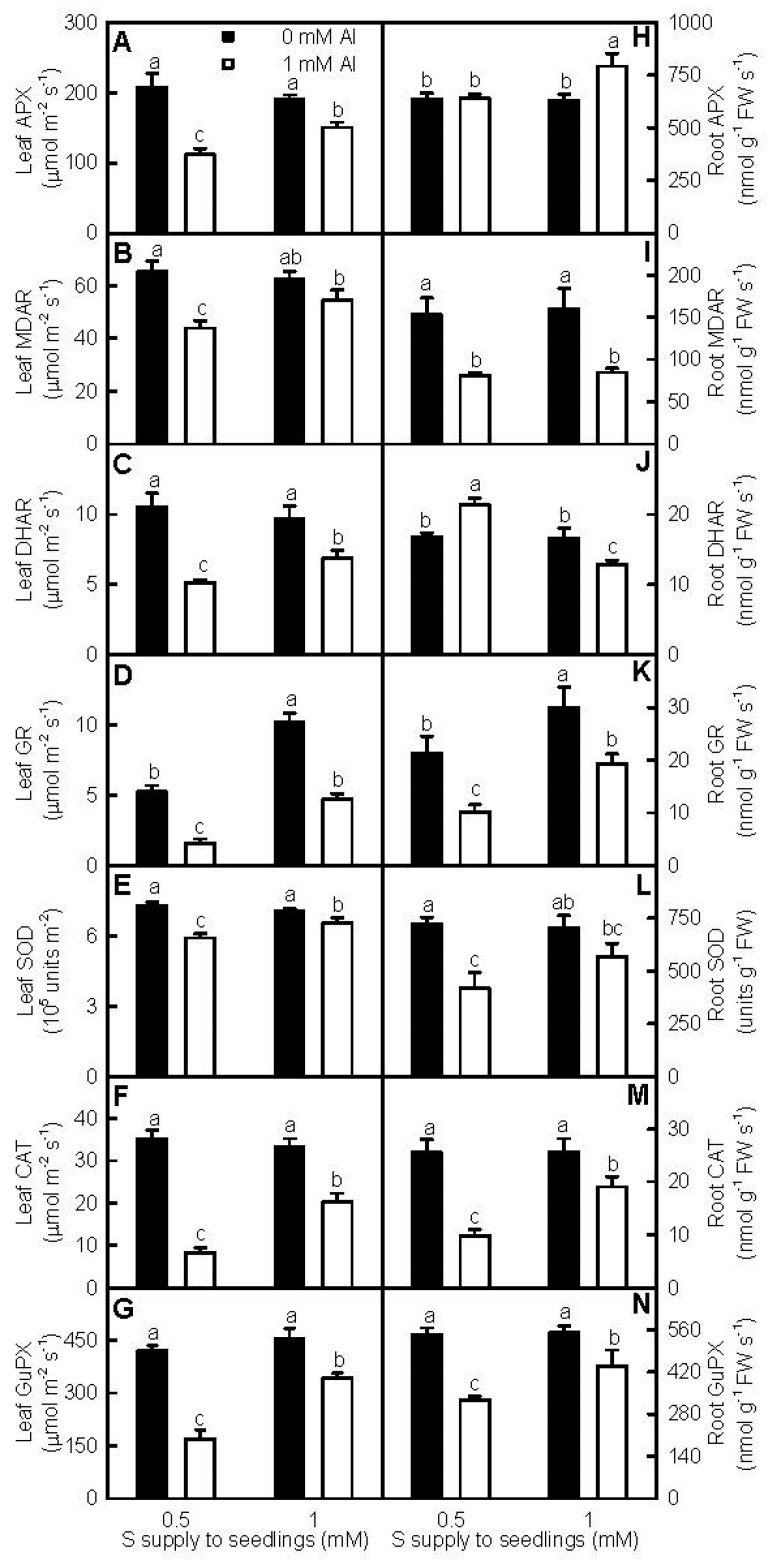
Effects of S and Al interactions on ascorbate peroxidase (APX, **A**,**H**), monodehydroascorbate reductase (MDAR, **B**,**I**), dehydroascorbate reductase (DHAR, **C**,**J**), glutathione reductase (GR, **D**,**K**), superoxide dismutase (SOD, **E**,**L**), catalase (CAT, **F**,**M**), and guaiacol peroxidase (GuPX, **G**,**N**) activities in leaves (**A**–**G**) and roots (**H**–**N**). Bars represent means ± SE (*n* = 4). Differences among the four treatments were analyzed by two (Al levels) × two (S levels) ANOVA. Different letters indicate a significant difference at *p* < 0.05.

We assayed the activities of root and leaf antioxidant enzymes (Figure 5), the first line of defense against the oxidative damage [27], in order to investigate whether antioxidant enzymes played a role in the S-mediated-alleviation of oxidative stress in Al-treated leaves and roots. In leaves, Al-toxicity decreased all the seven antioxidant enzyme activities, with the exception that monodehydroascorbate reductase (MDAR) activity was similar between the two Al treatments at 1 mM S. At the absence of Al, glutathione reductase (GR) activity was higher at 1 mM S than that at 0.5 mM S, and the other enzyme activities were similar between the two S treatments. Under Al-toxicity, the seven antioxidant enzyme activities were lower at 0.5 mM S than those at 1 mM S (Figure 5A–G). In roots, Al-toxicity decreased or did not affect the activities of all the seven antioxidant enzymes, with the exceptions that Al-toxicity increased ascorbate peroxidase (APX) activity at 1 mM S and dehydroascorbate reductase (DHAR) activity at 0.5 mM S. At the absence of Al, the activity of GR was higher at 1 mM S than thatat 0.5 mM S, and the activities of the other enzymes did not differ between the two S treatments. Under Al-toxicity, 1 mM S treatment decreased DHAR activity, did not affect MDAR activity, and increased the activities of the other five enzymes compared with 0.5 mM S treatment (Figure 5H–N).

**Figure 6:**
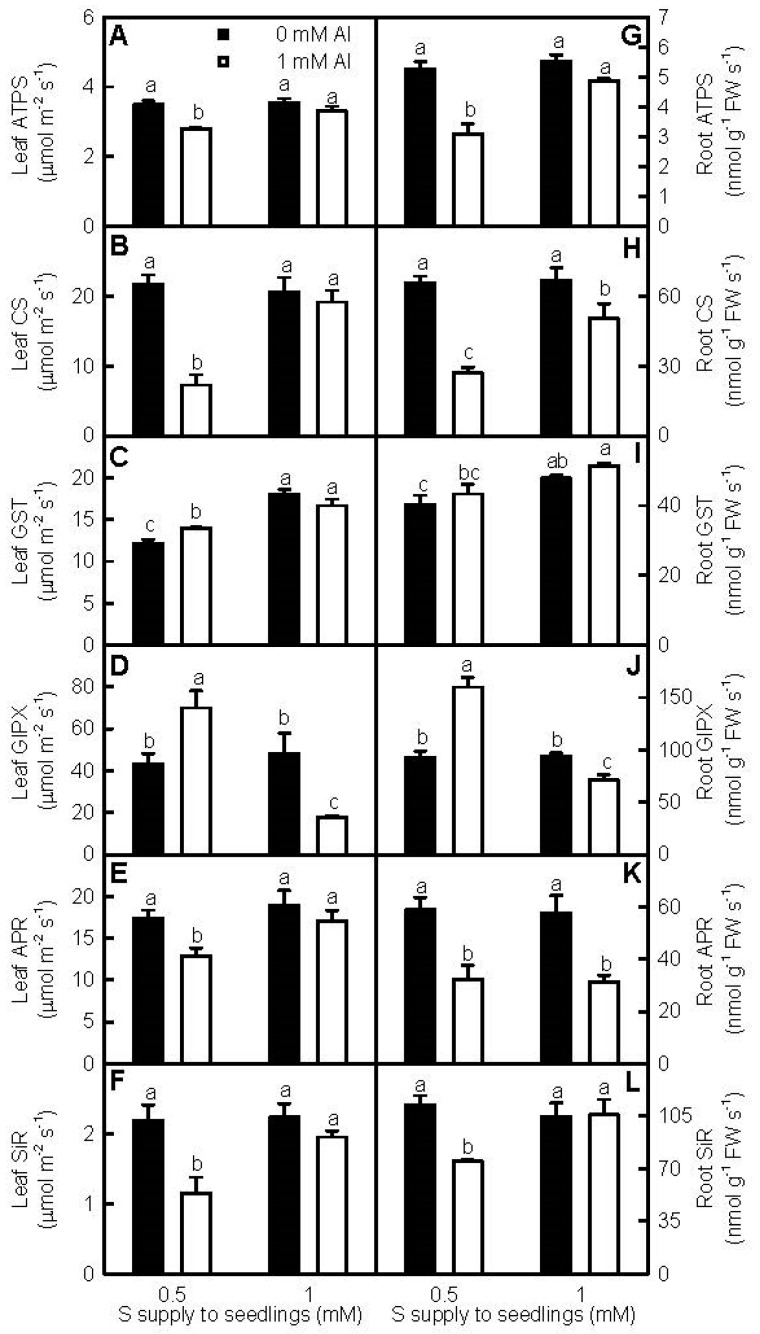
Effects of S and Al interactions on ATP sulfurylase(ATPS, **A**,**G**), cysteine synthase (CS, **B**,**H**), glutathione-S-transferase(GST, **C**,**I**), glutathione peroxidase (GlPX, **D**,**J**), adenosine 5′-phosphosulphate reductase (APR, **E**,**K**), andsulfite reductase (SiR, **F**,**L**) activities in leaves (**A**–**F**) and roots (**G**–**L**). Bars represent means ± SE (*n* = 4). Differences among the four treatments were analyzed by two (Al levels) × two (S levels) ANOVA. Different letters indicate a significant difference at *p* < 0.05.

The thiol-based antioxidant system is believed to be the second line of defense against the oxidative damage [27]. Al-toxicity decreased or did not affect the activities of the S metabolism-related enzymes in leaves and roots, with the exceptions that glutathione-S-transferase (GST) and glutathione peroxidase (GlPX) activities in 0.5 mM S-treated leaves, and GlPX activity in 0.5 mM S-treated roots were increased by Al-toxicity. At 0 mM Al, S did not affect ATP sulfurylase (ATPS), cysteine synthase (CS), GlPX, adenosine5′-phosphosulphate reductase (APR), and sulfite reductase (SiR) activities in leaves and roots, while GST activities in leaves and roots were higher at 1 mM S than those at 0.5 mM S. Under Al-toxicity, ATPS, CS, GST, APR, and SiR activities in leaves and roots were higher at 1 mM S than those at 0.5 mM S, with the exceptions that root APR activity did not differ between the two S treatments, while GlPX activities in leaves and roots were lower at 1 mM S than those at 0.5 mM S (Figure 6).

**Figure 7:**
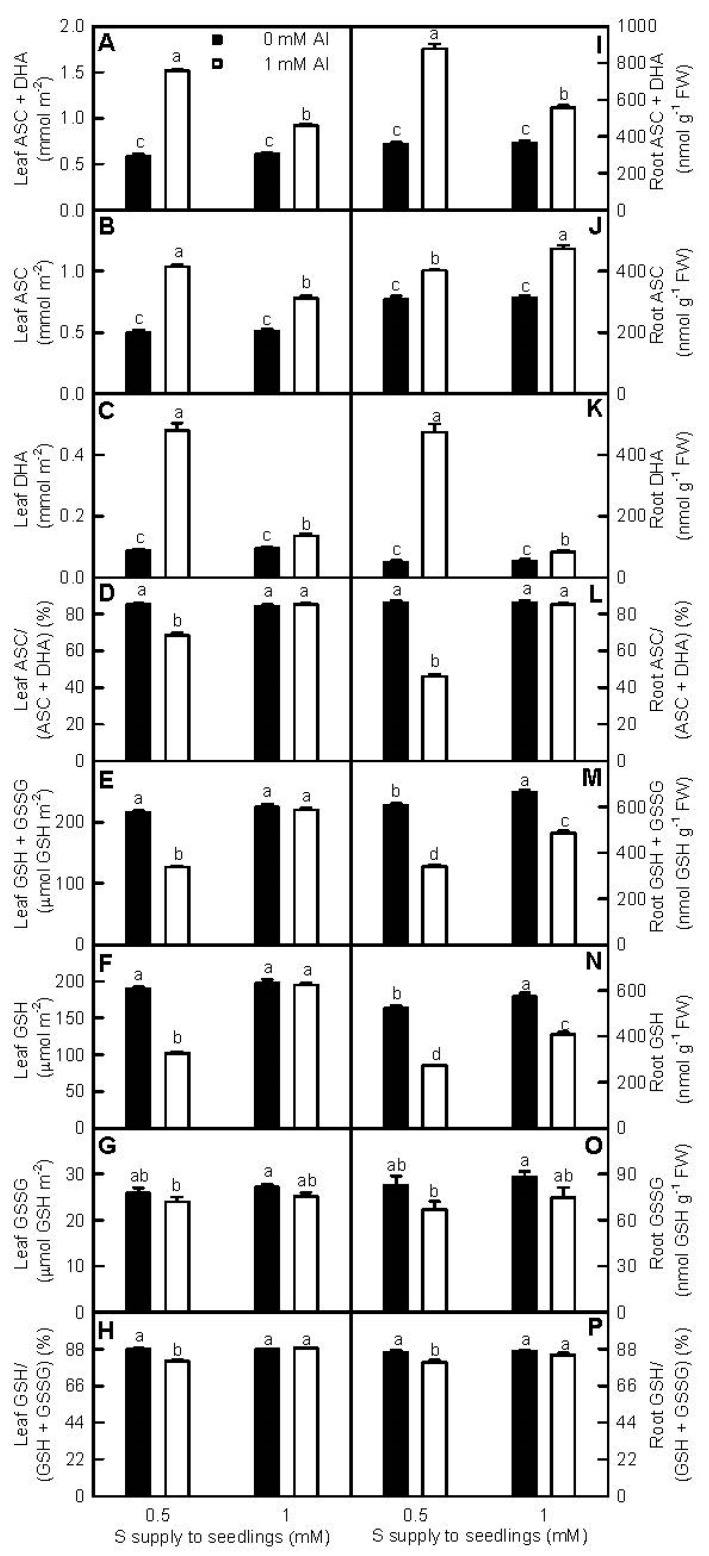
Effects of S and Al interactions onascorbate (ASC) +dehydroascorbate(DHA) (**A**,**I**), ASC (**B**,**J**), and DHA (**C**,**K**) concentrations; ASC/(ASC + DHA) ratios (**D**,**L**),reduced glutathione (GSH) +oxidized glutathione (GSSG) (**E**,**M**), GSH (**F**,**N**) and GSSG (**G**,**O**) concentrations; and GSH/(GSH + GSSG) ratios (**H**,**P**) in leaves (**A**–**H**) and roots (**I**–**P**). Differences among the four treatments were analyzed by two (Al levels) × two (S levels) ANOVA. Bars represent means ± SE (*n* = 4). Different letters indicate a significant difference at *p* < 0.05.

Meanwhile, we also measured the concentrations of ascorbate (ASC), dehydroascorbate (DHA), GSH, and oxidized glutathione (GSSG), the important small molecular antioxidants, in roots and leaves treated with different S and Al levels (Figure 7). Al-toxicity increased the levels of ASC + DHA, ASC, and DHA, decreased or did not affect the levels of GSH + GSSG, GSH, and GSSG and the ratios of GSH/(GSH + GSSG) and ASC/(ASC + DHA) in roots and leaves. At 0 mM Al, all these parameters in leaves and roots were similar between the two S treatments, with the exceptions that GSH + GSSG and GSH levels were slightly higher at 1 mM S than those at 0.5 mM S. Under Al-toxicity, ASC + DHA, ASC, and DHA levels in leaves, and ASC + DHA and DHA levels in roots were higher at 0.5 mM S than those at 1 mM S; while ASC level in roots was higher at 1 mM S than that at 0.5 mM S. The levels of GSH + GSSG and GSH, and the ratios of ASC/(ASC + DHA) and GSH/(GSH + GSSG) in leaves and roots were higher at 1 mM S than those at 0.5 mM S, while GSSG levels in leaves and roots were similar between the two S treatments.

## 3. Discussion

### 3.1. S Alleviated Al-Toxicity in C. grandis Seedlings by Decreasing the Transport of Al from Roots to Shoots and Increasing the Al-Induced Secretion of Citrate

We found that the Al-induced decreases in seedling growth, leaf CO_2_ assimilation, and pigments, and alterations of leaf OJIP transients and fluorescence parameters were more pronounced at 0.5 mM S than those at 1 mM S (Figure 1, Figure 3 and Figures S1–S4), demonstrating that S alleviated Al-toxicity in *C. grandis* seedlings. This was also supported by our data that the Al-induced increases of H_2_O_2_ production in roots and leaves and of electrolyte leakage in roots, and decreases of RWC and total soluble protein levels in roots and leaves were greater at 0.5 mM S than those at 1 mM S (Figure 4). In addition, the Al-induced increases of MDA level and electrolyte leakage in leaves (Figure 4H–I), and decreases of ASC/(ASC + DHA) and GSH/(GSH + GSSG) ratios in roots and leaves (Figure 7D,H,L,P) only occurred at 0.5 mM S. Plant Al-tolerance is related not only to low uptake of Al, but also to the relatively little translocation of Al from roots to shoots (leaves) [28]. Previous studies showed that P, boron (B), and sodium nitroprusside (SNP) could alleviate Al-toxicity through increasing the formation of non-toxic Al complexes at the root surface and/or in the root tissues and decreasing Al transport from roots to shoots [29,30,31,32,33,34]. Here, the ratio of root DW/shoot DW in +Al seedlings was higher at 1 mM S than that at 0.5 mM S (Figure 1F), and Al level in +Al roots did not differ between the two S treatments, but in +Al leaves was higher at 0.5 mM S than that at 1 mMS (Figure 2A,D), indicating that 1 mM S-treated seedlings increased the accumulation of Al in roots and decreased the transport of S from roots to shoots (leaves) compared with the 0.5 mM S-treated seedlings, thus alleviating Al-toxicity. However, NaHS-pretreatment could mitigate the Al-induced increases of Al levels in barley leaves and roots [9,23]. Thus, it appears that the effects of S on Al levels in roots and leaves and the transport of Al from roots to shoots depend on plants species. The Al-induced secretion of OA anions is an important mechanism of external Al detoxification. OA anions secreted by roots can chelate the Al, thus preventing Al uptake and the transport of Al from roots to shoots [2,8,23]. Chen et al. suggested that the S-mediated-alleviation of Al-toxicity involved the NaHS-dependent increase of citrate release in barley seedlings [23]. We found that the Al-induced secretion of citrate from +Al excised roots was higher at 1 mM S than that at 0.5 mM S (Figure S5B), implying that the stimulatory action of S on the Al-induced secretion of citrate might play a role in the S-mediated alleviation of Al-toxicity. However, the Al-induced release of malate from +Al excised roots was similar between the two S treatments (Figure S5A). Further studies are needed to answer this question.

### 3.2. Increased Leaf and Root RWC, and P, Ca, and Mg Contents per Plant Might Play a Role in the S-Mediated Alleviation of Al-Toxicity

Al-toxicity can damage the root system and interfere with the uptake of water and nutrients [2,9,32,35,36]. Under continuous drought stress, soybean seedlings sprayed with NaHS displayed higher survival rate and leaf and root RWC than non-sprayed controls did [37]. NaHS pretreatment prior to salinity and non-ionic osmotic stress greatly mitigated leaf Gs and RWC decreases in stressed strawberry plants [38]. Thus, the Al-induced inhibition of water uptake might be alleviated by S. As expected, the Al-induced decreases of root and leaf RWC were less at 1 mM S than those at 0.5 mM S (Figure 4A,F), although the transpiration rate in +Al leaves was higher at 1 mM S than that at 0.5 mM S (Figure S2D). The might be related to the finding that root DW was decreased by Al-toxicity at 0.5 mM S, and that the root DW/shoot DW ratio in +Al seedlings was higher at 1 mM S than that at 0.5 mM S (Figure 1A,F). Exposure to 1 mM Al led to significant decreases in P, Ca, and Mg levels in roots and leaves, with the exceptions that Mg level in 0.5 mM S-treated roots increased and Ca level in 1 mM S-treated roots did not change under Al-toxicity (Figure 2C–E,H–J). Obviously, Al-toxicity altered P, Ca, and Mg distribution in roots and leaves and their contents per plant, which might be responsible for nutrient deficiencies or imbalances and the inhibition of seedling growth. Because P, Ca, and Mg levels in Al-treated roots and leaves were higher at 1 mM S than those at 0.5 mM S or similar between the two S treatments (Figure 2C–E,H–J), their contents per plant under Al-toxicity should be higher at 1 mM S than those at 0.5 mM S because Al-treated seedlings displayed higher root, leaf, and shoot DW at 1 mM S than those at 0.5 mM S, and similar stem DW between the two S treatments (Figure 1A–D). Our findings that Al-treated seedlings had higher root and leaf DW at 1 mM S than those at 0.5 mM S, meaning that P and Mg were less diluted at 0.5 mM S than at 1 mM S. This can explain why Mg and P levels in Al-treated roots and leaves were similar between the two S treatments. Similar results have been obtained by Dawood et al. [9], who observed that the addition of 200 and 400 μM NaHS to Al solution elevated P, Ca, and Mg contents per plant, and increased plant height and biomass compared with Al-alone treatment, but did not significantly affect root P and Ca levels and shoot Mg levels. Previous studies showed that the toxic effects of Al on higher plants could be alleviated by the application of Ca, Mg, and P [32,36,39,40]. Thus, the increased contents of P, Ca, and Mg per plant in 1 mM S + 1 mM Al-treated seedlings compared with 0.5 mM S + 1 mM Al-treated seedlings might play a role in the S-mediated-alleviation of Al-toxicity. This agrees with the previous report that enhanced P, Mg, and Ca contents per plant might be one of the ameliorative mechanisms of NaHS to Al-toxicity in barley seedlings [9].

### 3.3. Possible Causes for S-Mediated-Alleviation of CO_2_ Assimilation Decline in Al-Treated Leaves

We observed that S alleviated the Al-induced inhibition of CO_2_ assimilation (Figure S2A), as found for Al-stressed barley [9]. Although Gs in +Al leaves was higher at 1 mM S than that at 0.5 mM S, the higher CO_2_ assimilation in +Al leaves at 1 mM S could not be explained alone by the elevated Gs, because C_i_ in +Al leaves was similar between the two S treatments (Figure S2B,C). This was also supported by the report that both CO_2_ assimilation and Gs in Al-stressed barley leaves were enhanced by NaHS, while the reverse was the case for C_i_ [9].

The Al-induced decrease in Rubisco activity has been suggested to not be the primary factor limiting CO_2_ assimilation in *Citrus* leaves [32,33,41,42]. Here, the ameliorative action of S against inhibitory effect of Al-toxicity on CO_2_ assimilation might also be not due to the elevated Rubisco activity, because CO_2_ assimilation was decreased more than Rubisco activity in +Al leaves (Figure S2A,E). Similarly, the alleviation of S on the Al-induced inhibition of photosynthesis was not due to increased Chl level because Al-toxicity affected photosynthesis more than Chl (Figures S2A and S3A–C). This was also supported by our data that the Al-induced increases of DI_o_/RC, DI_o_/ABS and K_N_ in leaves were greater at 0.5 mM S than those at 1 mM S (Figure S4F–H).

Studies showed that the impaired electron transport capacity accompanied by the shortage of reducing equivalents were the main causes responsible for the inhibition of CO_2_ assimilation in Al-stressed *Citrus* leaves [42], and that B, P, and SNP alleviated the Al-induced impairments of the whole photosynthetic electron transport chain from the donor side of PSII to the reduction of PSI end acceptors, thus preventing the decline of CO_2_ assimilation [32,33,34]. We found that the Al-induced alterations of OJIP transients and all the 20 fluorescence parameters were less pronounced at 1 mM S than those at 0.5 mM S (Figure 3 and Figure S4). Therefore, we concluded that S prevented the Al-induced inhibition of photosynthesis via alleviating the Al-induced decrease of electron transport capacity due to the impaired photosynthetic electron transport chain.

### 3.4. In Addition to Decreasing Al-Induced ROS Production, S-Induced Upregulation of Both Antioxidant Enzymes and S Metabolism-Related Enzymes Played a Role in S-Mediated-Alleviation of Al-Toxicity

Our results clearly showed that S mitigated the Al-induced increases of H_2_O_2_ (ROS) production in roots and leaves (Figure 4B,G). ROS can cause damage to all cellular components [24]. As expected, Al-toxicity increased MDA level and electrolyte leakage in roots and leaves, with the exceptions that MDA level and electrolyte leakage in 1 mM S-treated leaves were not altered by Al-toxicity. The Al-induced damage on cell membrane integrity was alleviated by S, as indicated by the lower electrolyte leakage in +Al roots and leaves at 1 mM S than that at 0.5 mM S (Figure 4D,I). Interestingly, S mitigated the Al-induced lipid peroxidation in leaves, but not in roots because MDA concentration in +Al roots was similar between the two S treatments (Figure 4H). Based on these results, we concluded that S might lower the Al-induced accumulation of MDA, hence decreasing the damage of Al-toxicity on leaf cell membrane integrity. Also, S might play a direct role in maintaining root cell membrane integrity.

Plants have developed efficient enzyme and non-enzyme systems to eliminate oxidative damage [19,24,43]. Antioxidant enzymes, the first line of defense against the oxidative damage [27], have been shown to play a role in plant overall Al-tolerance [15,17]. Both transgenic tobacco plants overexpressing *Arabidopsis* cytosolic *DHAR* [44] and transgenic canola plants overexpressing wheat *manganese SOD* [45] displayed enhanced Al-tolerance. Qian et al. found that the Al-induced increases of MDA and H_2_O_2_ levels and decreases of APX, superoxide dismutase (SOD), guaiacol peroxidase (GuPX), catalase (CAT), and GR activities in *Brassica napus* leaves and roots were alleviated by NaHS [25]. H_2_S and SO_2_ mitigated Al-toxicity in germinating wheat seeds [16,24] and barley seedlings [9,23] via enhancing antioxidant capability, lessening oxidative stress, and maintaining membrane integrity. In this experiment, the activities of all the seven antioxidant enzymes in +Al roots and leaves were higher at 1 mM S than those at 0.5 mM S, with the exceptions that DHAR activity in +Al roots was higher at 0.5 mM S than that at 1 mM S, and that MDAR activity in +Al roots was similar between the two S treatments (Figure 5). The thiol-based antioxidant system is considered to the second line of defense against the oxidative stress [27]. S metabolism is a kernel pathway required for the biosynthesis of S-containing compounds. Through synthesizing S-containing compounds, ATPS and other enzymes in S metabolism play crucial roles in plant adaptation to unfavorable conditions, including Al-toxicity [22,46,47,48]. Transgenic *Arabidopsis* plants overexpressing *GST* displayed elevated Al-tolerance [47]. Yang et al. used two-dimensional electrophoresis (2-DE) to identify 12 proteins increased in abundance and five proteins decreased in abundance from Al-treated rice roots. Further analysis showed that CS played an important role in Al-tolerance [22]. Jiang et al. [18] and Li et al. [19] used isobaric tags for relative and absolute quantification (iTRAQ) to identify more S metabolism-related proteins increased in abundance in Al-treated Al-tolerant *C. sinensis* roots and leaves than those in Al-treated Al-intolerant *C. grandis* roots and leaves. Guo et al. [20] isolated more upregulated than downregulated or less upregulated than downregulated S metabolism-related genes from Al-stressed *C. sinensis* or *C. grandis* roots, respectively. We found that S prevented the Al-induced decreases of ATPS, CS, GST, APR, and SiR activities in roots and leaves, with the exception that APR activity in +Al roots was not altered by S. Interestingly, GlPX activities in 0.5 mM S-treated roots and leaves were elevated by Al-toxicity, and in +Al roots and leaves were lower at 1 mM S than those at 0.5 mM S (Figure 6). The main reaction that GlPX catalyzes is: 2GSH + H_2_O_2_ → GSSG + 2H_2_O, thus protecting cells against oxidative damage. Glutathione pool in transgenic tobacco plants overexpressing *GST*/*GlPX* was more oxidized than that in wild-type plants [49,50]. In addition to meeting the increased requirement for scavenging the Al-stimulated production of H_2_O_2_, the Al-induced increases of GlPX activities in 0.5 mM S-treated leaves and roots might contribute to the lower GSH/(GSH + GSSG) ratios in +Al leaves and roots (Figure 7H,P). In 1 mM S-treated roots and leaves, Al-toxicity decreased GlPX activities (Figure 6D,K), but did not affect GSH/(GSH + GSSG) ratios (Figure 7H,P). Other factors might play a role in regulating the oxidized status of glutathione pool. Cellular redox homeostasis is an indispensable buffering mechanism that avoids excessive oxidation or reduction [51]. Our results showed that the Al-induced decreases of both ASC/(ASC + DHA) and GSH/(GSH + GSSG) ratios were greater at 0.5 mM S than those at 1 mM S (Figure 7D,H,L,P). Dixit et al. suggested that the S-mediated-alleviation of oxidative stress involved in both efficient thiol metabolism and antioxidant systems in arsenic-treated rice [52]. Thus, we concluded that S decreased the Al-induced production of ROS and the damage of Al-toxicity on cell membrane integrity, and prevented the Al-induced decreases in the activities of both antioxidant enzymes and S metabolism-related enzymes and the ratios of both ASC/(ASC + DHA) and GSH/(GSH + GSSG) in leaves and roots, thus leading to the mitigation of Al-toxicity.

## 4. Materials and Methods

### 4.1. Seedling Culture and Treatments

‘Shatian pummelo’ (*C. grandis*) seeds were collected from Meizhou Academy of Agricultural Sciences, Meizhou, Guangdong, China. Seedling culture and Al treatments were made according to Guo et al. [20] and Zhou et al. [53]. Five weeks after sprouting, uniform ‘Shatian pummelo’ (*C. grandis*) seedlings were transplanted to 6 L pots (two seedlings per pot) filled with river sand, then grown in a greenhouse with natural photoperiod at Fujian Agriculture and Forestry University (FAFU), Fuzhou (26°5′ N, 119°14′ E). One week after transporting, each pot was irrigated with 500 mL of nutrient solution per two days. The nutrient solution contained 1 mM KNO_3_, 1 mM Ca(NO_3_)_2_, 0.1 mM KH_2_PO_4_, 0.5 mM MgSO_4_, 10 μM H_3_BO_3_, 2 μM MnCl_2_, 2 μM ZnSO_4_, 0.5 μM CuSO_4_, 0.065 μM (NH_4_)_6_Mo_7_O_24_, and 20 μM Fe-EDTA. Six weeks after transplanting, each pot was irrigated daily until dripping (~500 mL) with the above nutrition solution containing 0 or 0.5 mM Na_2_SO_4_ and 0 (−Al) or 1 mM (+Al, Al-toxicity) AlCl_3_·6H_2_O. Seedlings that did not receive Na_2_SO_4_and AlCl_3_·6H_2_O were used as controls. Total S concentration in the solution was 0.5 or 1 mM. The pH of the solution was adjusted to 4.1–4.2 with HCl or NaOH. No precipitates were formed in the nutrient solutions. Al concentrations in nutrient solutions containing 0 (0.945 ± 0.039 mM) and 0.5 (0.955 ± 0.038 mM) mM Na_2_SO_4_were similar. The concentration of 0.5 mM Na_2_SO_4_ was selected for this study based on our preliminary study. In this study, we investigated the antagonistic actions of 0, 0.5, and 1.5 mM Na_2_SO_4_ against the inhibitory effects of 1 mM Al on ‘Shatian pummelo’ seedling growth and photosynthesis. The order of Na_2_SO_4_ effectiveness was 0.5 mM > 1.5 mM > 0 mM. Eighteen weeks after Al treatments, ~5-mm-long white root apices and recent fully expanded mature leaves were chosen for all the measurements except for root elements. After Chl a fluorescence and gas exchange were determined, leaf discs (0.6 cm in diameter) and ~5-mm-long white root apices from the same seedlings were collected on a sunny noon and frozen in liquid N_2_, then stored at −80°C until use for the measurements of enzymes, antioxidants, MDA, and total soluble proteins. The unsampled seedlings were used to measure biomass, Al-induced secretion of OA anions from roots, electrolyte leakage, RWC, H_2_O_2_ production, and elements in roots and leaves.

### 4.2. Biomass, Leaf Pigments, and Root and Leaf Total Soluble Proteins and Elements

Fifteen seedlings per treatment from 15 pots were collected. Root, stem, and leaf DW were measured after being dried to a constant weight at 70 °C (~48 h).

Chl a, Chl b, and Car were assayed according to Lichtenthaler [54]. Total soluble proteins were determined according to Bradford [55].

Fibrous roots and recent fully expanded mature leaves (midribs, petioles, and winged leaves removed) were collected. P, S, and Al were measured using the ammonium molybdate–ascorbic acid spectrophotometric assay [56], the simple turbidimetric method [57], and the aluminon method [58], respectively. Ca and Mg were measured with a PinAAcle 900F Atomic Absorption Spectrometer (Perkinelmer Singapore Pte Ltd., Singapore).

### 4.3. Root and Leaf Electrolyte Leakage, RWC, H_2_O_2_ Production and MDA

Electrolyte leakage, RWC, and H_2_O_2_ production were measured according to Long et al. [57]. MDA was determined according to Hodges et al. [59].

### 4.4. Al-Induced Secretion of Malate and Citratefrom Roots

Al-induced secretion of malate and citrate was assayed according to Yang et al. [28].

### 4.5. Antioxidant Enzymes and S Metabolism-Related Enzymes in Leaves and Roots

Leaf and root GuPX, SOD, APX, MDAR, DHAR, GR, CAT, GlPX, and GST were extracted with 50 mM KH_2_PO_4_-KOH (pH 7.5) containing 0.1 mM EDTA-Na_2_, 0.3% (*w*/*v*) Triton X-100, and 4% (*w*/*v*) insoluble polyvinylpolypyrrolidone (PVPP) [43]. ATPS and CS were extracted with 20 mM Tris-HCl (pH 8.0) buffer containing 10 mM EDTA-Na_2_, 2 mM dithiothreitol, and 4% (*w*/*v*) insoluble PVPP [60]. APR and SiR were extracted with 100 mM Tris-HCl (pH 8.0) buffer containing 10 mM EDTA-Na_2_ and 5% (*w*/*v*) PVPP [61,62]. GuPX and SOD were assayed according to Chen et al. [63] and Giannopolitis and Ries [64], respectively. APX, CAT, MDAR, DHAR, and GR were measured according to Chen and Cheng [43]. GlPX and GST were assayed according to Hasanuzzaman et al. [65] and Fujita and Hossain [66], respectively. ATPS was assayed according to Lappartient and Touraine [60]. One hundred μL of enzyme extract was incubated for 15 min at 37 °C with 0.5 mL of reaction mixture, which contained 80 mM Tris-HCl buffer (pH 8.0), 7 mM MgCl_2_, 2 mM Na_2_ATP, 5 mM Na_2_MoO_4_, and 0.032 U·mL^−1^ of sulfate-free inorganic pyrophosphatase. Then, phosphate was determined according to Ames [56]. CS, APR, and SiR were assayed according to Warrilow and Hawkesford [67], Trüperand Rogers [62], and Ostrowski et al. [61], respectively.

### 4.6. GSSG, GSH, DHA, and ASC in Leaves and Roots

Leaf and root GSH and GSSG were measured according to Griffith [68] after being extracted with 5% (*w*/*v*) of trichloroacetic acid. Leaf and root ASC and DHA were assayed according to Chen et al. [69] after being extracted with 6% (*v*/*v*) of HClO_4_.

### 4.7. Leaf Gas Exchange and Rubisco

Leaf gas exchange was measured with a CIARS-2 portable photosynthesis system (PP systems, Herts, UK) at an ambient CO_2_ concentration under a controlled light intensity of ~1000 μmol·m^−2^·s^−1^ between 9:00 and 11:00 on a sunny day. Rubisco activity was measured according to Long et al. [57].

### 4.8. Leaf OJIP Transients by Handy PEA

Leaf OJIP transients were measured with a Handy PEA (Hansatech Instruments, Norfolk, UK) after seedlings being dark-adapted for 3 h. The following extracted data from the original measurements were used: fluorescence intensities at 20 μs (F_20μs_, considered as minimum fluorescence F_o_), 50 μs (F_50μs_), 300 μs (F_300μs_), 2 ms (J-step), 30 ms (I-step), and P-step (considered as maximum fluorescence F_m_). The fluorescence parameters—maximum variable fluorescence (F_v_), approximated initial slope (in ms^−1^) of the fluorescence transient V = f(t) (M_o_), specific energy fluxes per reaction center (RC) for absorption (ABS/RC), and energy dissipation (DI_o_/RC); maximum quantum yield of primary photochemistry (F_v_/F_m_), quantum yield for the reduction of end acceptors of photosystem I (PSI) per photon absorbed (φ_Ro_), energy dissipation (φ_Do_), and electron transport (φ_Eo_); photochemical (K_P_) and non-photochemical (K_N_) deexcitation rate constants, overall grouping probability (P_2G_), performance (PI_abs_), and total performance (PI_tot,abs_) indexes—were calculated according to Chen and Cheng [70], Jiang et al. [42], Liao et al. [71], Srivastava et al. [72], and Strasser et al. [73].

### 4.9. Leaf Chl a Fluorescence Parameters by FMS-2

Leaf Chl a fluorescence was determined with a pulse-modulated fluorometer FMS-2 (Hansatech Instruments, Norfolk, UK). Photochemical quenching coefficient (qP), actual quantum of PSII electron transport (Φ_PSII_), efficiency of excitation transfer to PSII RCs under natural light (F_m_′/F_v_′), and electron transport rate through PSII were calculated according to Long et al. [57] and Genty et al. [74].

### 4.10. Statistical Analysis

There were 20 pot seedlings (replicates) per treatment in a completely randomized design. Experiments were performed with 3–18 replicates. Differences among the four treatments were analyzed by two (Al levels) × two (S levels) ANOVA; the four means were separated by the Duncan’s new multiple range test at *p* < 0.05 level.

## 5. Conclusions

Our results clearly demonstrated that S alleviated the Al-induced inhibition of growth and photosynthesis in *C. grandis* seedlings via decreasing the transport of Al from roots to shoots and relatively little leaf Al accumulation, and increasing the Al-induced secretion of citrate by roots. In addition to decreasing the Al-stimulated ROS production, the upregulation of both S metabolism-related enzymes and antioxidant enzymes were also responsible for the S-mediated-alleviation of oxidative stress in +Al roots and leaves. Under Al-toxicity, root and leaf RWC, and P, Ca, and Mg contents per plant were higher in 1 mM S-treated seedlings than those in 0.5 mM S-treated seedlings. Increased root and leaf RWC, and P, Ca, and Mg contents per plant might also play a role in the S-mediated-alleviation of Al-toxicity. To conclude, our findings support the hypothesis that S may alleviate *Citrus* Al-toxicity.

## Supplementary Materials

Supplementary materials can be found at www.mdpi.com/1422-0067/18/12/2570/s1.
