# Supplementary material for: Sulfur-Mediated-Alleviation of Aluminum-Toxicity in Citrus grandis Seedlings
Source: Int J Mol Sci. 2017 Dec 3;18(12):2570. doi: 10.3390/ijms18122570 (PMC5751173; doi:10.3390/ijms18122570)
Supplement: Supplementary file 1 [file ijms-18-02570-s001.pdf]

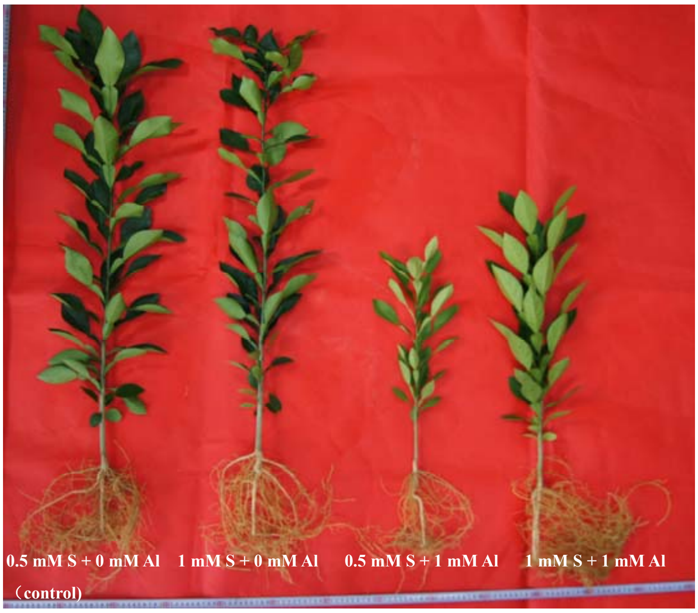

Supplementary Figure S1. Effects of S and Al interactions on *C. grandis* seedling growth.

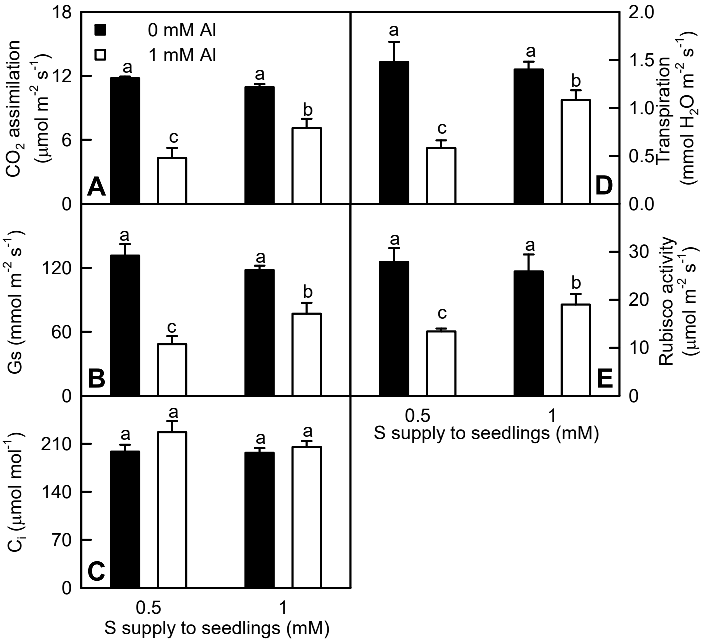

Supplementary Figure S2. Effects of S and Al interactions on leaf gas exchange and Rubisco activity. Bars represent means ± SE (n = 3 - 5). Differences among four treatments were analyzed by two (Al levels) × two (S levels) ANOVA. Different letters indicate a significant difference at $P < 0.05$.

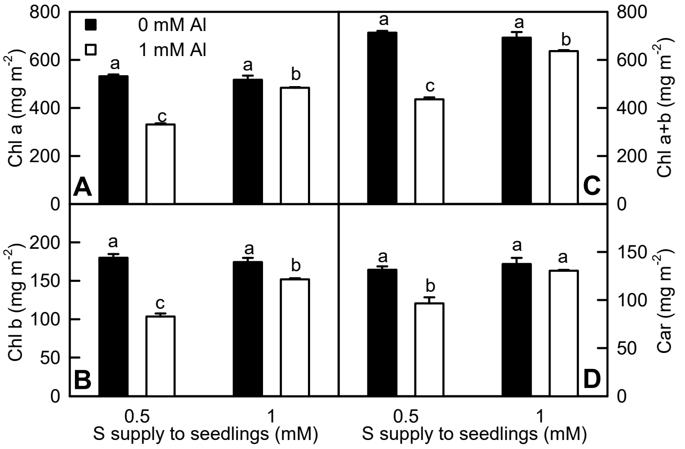

Supplementary Figure S3. Effects of S and Al interactions on leaf pigments. Bars represent means ± SE (n = 4). Differences among four treatments were analyzed by two (Al levels) × two (S levels) ANOVA. Different letters above the bars indicate a significant difference at P < 0.05.

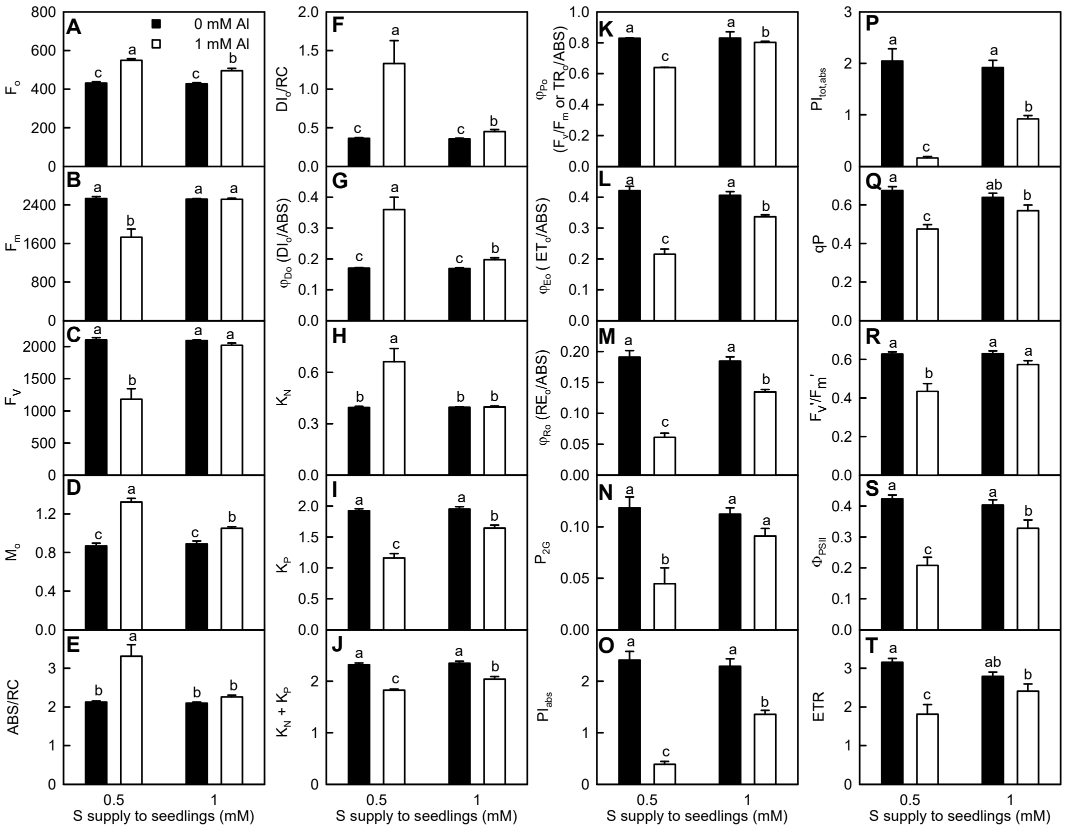

Supplementary Figure S4. Effects of S and Al interactions on 20 fluorescence parameters in leaves. Bars represent means ± SE (n = 4 - 18). Differences among four treatments were analyzed by two (Al levels) × two (S levels) ANOVA. Different letters above the bars indicate a significant difference at P < 0.05.

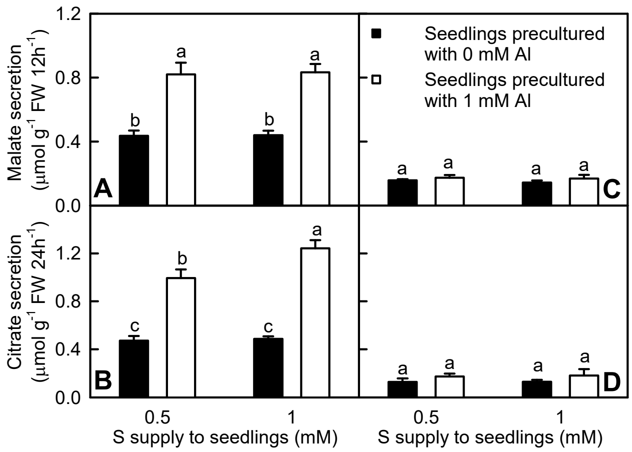

Supplementary Figure S5. Al-induced secretion of malate and citrate by excised roots from seedlings submitted to different S and Al levels for 18 weeks. Malate and citrate secretion from excised roots was measured after 12 or 24 h incubation, respectively in 0.5 mM $CaCl_2$ + 0.5 mM $AlCl_3 \bullet 6H_2O$ (**A** and **B**) or 0.5 mM $CaCl_2$ (**C** and **D**) solution, pH 4.1-4.2. Bars represent means ± SE (n = 4). Differences among four treatments were analyzed by two (Al levels) × two (S levels) ANOVA. Different letters above the bars indicate a significant difference at $P < 0.05$.
